# Phages in the fight against COVID-19?

**DOI:** 10.2217/fmb-2020-0082

**Published:** 2020-08-26

**Authors:** Andrzej Górski, Ryszard Międzybrodzki, Maciej Żaczek, Jan Borysowski

**Affiliations:** ^1^Bacteriophage Laboratory & Phage Therapy Unit, Institute of Immunology & Experimental Therapy, Polish Academy of Sciences, 53–114 Wroclaw, Poland; ^2^Department of Clinical Immunology, The Medical University of Warsaw, 02–006 Warsaw, Poland; ^3^Department of Clinical Immunology, Infant Jesus Clinical Hospital, 02–006 Warsaw, Poland

**Keywords:** coronavirus, COVID, phage, phage therapy

The COVID-19 pandemic has overwhelmed healthcare systems, putting staff under stress and bringing unprecedented challenges to the economy and social life [1]. At least 15% of COVID-19 patients suffer from severe disease, so effective treatment is desperately needed. The international scientific community has engaged in many studies to find drugs to treat the disease and there are reports suggesting the potential efficacy of some agents. However, it must be emphasized that although “*many drugs have in vitro activity against different coronaviruses, no clinical evidence currently supports the efficacy and safety of any drug in humans*” [2]. At the same time, the progress of clinical investigation is under threat as the pandemic shuts down research on drugs for multiple other diseases, including cancer, where, as some experts believe, clinical studies have been cut to almost zero [3]. In this extreme situation repurposing drugs may be the appropriate strategy to seek a prompt and hopefully efficient therapeutic response to the COVID-19 pandemic. A good example of such a strategy is the current enthusiasm for repurposing metformin, a well-known drug used for the treatment of diabetes Type 2, for the prevention and treatment of cancer [4]. There have also been attempts to repurpose metformin for cardiovascular diseases [5], and even for bacterial infections [6].

As mentioned, a number of drugs are currently being studied for their ability to be repurposed as a treatment for COVID-19 (chloroquine, azithromycin, lopinavir–ritonavir). These drugs may cause significant side effects, such as cardiac and hepatic toxicity, pancreatitis, bone marrow toxicity, etc., yet their clinical efficacy has not been proven so far – no ‘magic pill’ is currently available. Therefore, there is an urgent need for other agents that could be repurposed to combat the epidemic [7].

The present antibiotic crisis has greatly ignited interest in the potential of phages in treating multidrug-resistant bacterial infections with the aid of phage therapy. Hopefully, the current trend toward a personalized medicine may help to introduce the therapy in modern clinical medicine [8]. On the other hand, recent progress in our understanding of phage immunobiology opens perspectives for the repurposing of phage therapy to clinical indications other than bacterial infections alone. There are data suggesting that such indications could also include viral infections, possibly including COVID-19.

In 2005 we published a short review summarizing available historical data strongly suggesting that phages, both *in vitro* and *in vivo*, may interfere with eukaryotic viruses. These studies were initiated some half a century ago and pointed to antiviral effects of intact phages, phage lysates, as well as phage-derived nucleid acids. Such antiviral activities of phages could depend on phage-induced inteferon production, competition of phages and eukaryotic viruses for the same cellular receptors, as well as induction of antiviral antibodies cross-reacting with pathogenic viruses (phage vaccines). In addition, phage-mediated inhibition of other phenomena relevant in virus-dependent pathology may also be involved [9].

The results of research carried out in the following 15 years support the assumption that studies focusing on repurposing phage therapy at least as an adjunct therapy for viral infections, are warranted.

## Phages may penetrate epithelial cells, protect those epithelial & myeloid cells from virus-induced apoptosis & regulate expression of protective cellular chaperones

We demonstrated protective activity of the T4 phage on the survival of human lung epithelial cells infected with human Adv when the phage was added before or during the entire time of Adv infection; preincubation with the phage was also protective [10]. Interestingly, while SARS-CoV and SARS-CoV-2 are known to induce apoptosis and resulting lymphocytopenia is a common laboratory finding (>60 of patients and >80% in the dead patients) [11,12], apoptosis is reduced in airway epithelial cells harvested from the human bronchi and cultured with phages *in vitro* [13]. Furthermore, both T4 and A3/R (staphylococcal phage) preparations significantly reduce the percentage of DEC-205+ human myeloid cells (lectin receptor recognizing ligands expressed during apoptosis and necrosis of different cell populations) [14]. Both T4 and A5/80 phages may cause a high overexpression of the *HSA1* gene coding a heat shock protein, Hsp70, in human alveolar cells [15]. This well-known cellular chaperone may protect cells undergoing transcytosis from potential injury by cellular phages [16]. On the other hand, expression of another heat shock protein Hsp90, may be downregulated by T4 and M13 phages [17]. Hsp90 is relevant for the life cycle of viruses and its inhibitors may have antiviral properties [18,19].

We hypothesized that phages present in the human body may transmigrate from the gut and mediate immunomodulatory activities at different tissue sites [20]. This hypothesis has been fully confirmed by recent studies that have revealed that phages present in the human body may penetrate epithelial cell layers from gut, lung and other organs without causing any harmful effects. For example, T4 phage can transcytose across epithelial cells and this phenomenon encompasses diverse phages from major morphotypes and Gram-positive and negative hosts. The average human body transcytoses 3.1×10^10^ phages per day and it is believed that this continuous stream of endogenous phages disseminating through the blood and organs may provide antimicrobial defenses [21,22]. Furthermore, a comparable load of phages may be infused to patients during phage therapy [23]. Thus, phages infused during phage therapy provide significant additional loads of circulating phages, able to permeate epithelial cells in a process described by Nguyen *et al.* [21]. In other words, an increase in phage transcytosis by epithelial cells caused by phage therapy could build a protective barrier to eukaryotic virus particles. Since lung epithelium is also engaged in transcytosis of phages [21], this phenomenon could play some role in protecting those cells from invasion by coronaviruses.

## Inhibitory effects of phages on viral adsorption to human epithelial cells & viral replication

It was shown that the T4 phage inhibits adsorption of human Adv to human lung and kidney epithelial cells; moreover, viral replication was also inhibited [24]. T4 phage could also impair CoV attachment to target cells through its KGD sequence. This integrin KGD motif (Lys-Gly-Asp) present in the capsid protein of the T4 phage is functional and may mediate interactions of phage with eukaryotic cells [16]. KGD is found prominently on gH/gl glycoprotein orchestrating infection of EBV of B cells and epithelial cells, and its inactivation downregulates EBV interaction with target cells [25]. Therefore, T4 could interfere with EBV infection [26]. Interestingly, this amino acid sequence is conserved in several coronaviruses and it is also displayed in exposed loop of major structural SARS-CoV protein S. Moreover, the same KGD sequence is also expressed in angiotensin-converting enzyme 2 molecule, a recognition receptor for protein S, suggesting a potential inhibitory role for integrins in the receptor targeting of SARS-CoV and SARS-CoV-2 [27]. Therefore, T4 phage could also compete with coronaviruses and angiotensin-converting enzyme 2 binding and thereby inhibit coronavirus infection of target cells.

Both T4 and A5/80 antistaphylococcal phages significantly reduced the expression of HAdV genes while synthesis of HAdV DNA was inhibited only by the T4 phage [28].

## Anti-inflammatory effects of phages: phage-dependent inhibition of NF-κB & ROS production

Pathogenic viruses (including coronaviruses) induce NF-κB transcription factor upregulating the expression of genes involved in immune response [11]. Preincubation of endothelial and epithelial cells with the T4 phage can abolish or significantly reduce NF-κB activation triggered by herpes virus [29]. Interestingly, Zhang *et al.* [30]. reported similar effects using an antistaphylococcal phage.

Lung infection with respiratory viruses is associated with inflammation and cell death caused by excessive reactive oxygen species (ROS) production and the correlation between severity of lung injury and the markers of oxidative stress was noted in patients infected with the human respiratory syncytial virus [31]. Moreover, cellular ROS are markedly increased in SARS-CoV expressing cells [11], while high levels of ROS associated with a decrease in chest radiographic scores are observed early in COVID-19 [32]. Phages are known to downregulate ROS production [9]. Many studies point to the positive role of anti-oxidant therapy in infected cells and animals; however, there are almost no clinical data [31]. Nevertheless, anti-oxidant supplementation was recommended for the treatment of COVID-19 [33,34].

## Phages as potential inducers of antiviral immunity

There are also data suggesting that phages may drive antiviral immunity by inducing antiviral cytokines, for example, IFN -α and IL-12. Already in 1977, Taborsky and Dolnik showed that phage RNA may induce IFN-α in human granulocytes [35]. Recently, Sweere e*t al*. demonstrated that Pf phages (and phage RNA) endocytosed by leukocytes trigger TLR3-dependent pattern recognition receptors and inhibit TNF-driving type I IFN production [36]. In the absence of bacterial infection, the *Escherichia coli* phage 536_PI promotes an increase in the production of IFN-α and IL-12 in the lungs (but not in the blood), allowing for the presence of an antiviral signature in the lungs of healthy uninfected mice (this effect was phage specific and mediated by 536_P1 and not LM33 P1 phages) [37]. This phage-dependent antiviral signature in the lungs may occur since phages have the ability to penetrate this organ through multiple routes, so phage therapy has been used successfully in bacterial infections of the lungs in animals and humans [38]. Interestingly, a healthy respiratory virome includes phages, while the incidence of viral pathogens (including CoV) is associated with a reduced percentage of phages [39]. Recent data by Gogokhia *et al.* indicate that Lactobacillus, *E. coli* and Bacteroides phages and phage DNA may stimulate IFN-γ production via TLR9 activation [40]. IFN-γ is another potent antiviral cytokine [41].

TNF produced during inflammation is relevant for the coordinated development of the inflammatory response. However, excessive TNF production can cause an increased risk of viral replication and bacterial infections. Therefore, a therapeutic agent whose action could regulate TNF production to keep it at levels optimal for patients would be particularly welcome. Preclinical studies suggest that experimental viral pneumonia may be ameliorated by anti-TNF therapy. As increased levels of TNF are demonstrable in blood and tissue samples from patients with COVID-19 the recent article in *The Lancet* calls for urgent trials of anti-TNF therapy in this disorder [42]. As mentioned [36] some phages may inhibit TNF production, which is confirmed by earlier data by other authors who showed that phages may downregulate TNF-α levels in serum and lungs of mice with experimental acute pneumonia [43,44]. Interestingly, clinical phage therapy may reduce TNF production when its pretreatment level is high and increase it in low responders [45].

Those data may be considered as a relevant argument for also considering phages as a potential agent that could help achieving and maintaining TNF levels, allowing for appropriate antiviral immune responses in COVID-19 while reducing the risks of excessive immunosuppression.

Phages may also interact with TLR. In this regard, we have shown that the T4 phage increases the expression of the *TLR2* gene [15]. TLR2 is involved in antiviral responses as a result of recognition of the repeating protein subunit patterns common to many viral capsids [46]. Other antiviral effects could be mediated by the A5/80 staphylococcal phage through its ability to increase the expression of the *IL-2* gene. IL-2 drives NK cell activity, which is important in the defense against viral infections [15].

Phages can also induce antiviral immunity by upregulating expression of defensin 2, we have recently shown that the T4 phage may induce a marked upregulation of gene coding for hBD2, a multifunctional peptide expressed mainly in epithelial cells with antiviral activity [47]. Viral inactivation by that peptide includes direct binding of the virus by hBD2, reduction of viral replication and modulation of signaling pathways necessary for antiviral effects, as well as recruitment of immune cells contributing to antiviral activity leading to downregulation of cytopathic effects in human alveolar and laryngeal epithelial cells [48]. Experimental studies in mice have revealed a direct link between beta-defensin expression and pulmonary immunity. Moreover, participation of hBD2 in antiviral defenses in the respiratory tract has been confirmed in human disorders [49].

It has been suggested that phages may be repurposed for the treatment of not only bacterial, but also nonbacterial infections including viral and fungal infections (Adv, Epstein–Barr virus, *Aspergillus fumigatus*, *Candida albicans*) [26,29]. It appears that there are grounds to believe that phages may be included among drugs currently being studied for repurposing in the treatment of COVID-19. Based on the above data the most likely application of phages in COVID-19 could be in an adjunct antiviral therapy, similar to the current trend to combine phages with antibiotic therapy in bacterial infections. In addition, standard phage therapy could be considered for the treatment of bacterial complications of COVID-19, which occur in >40% of patients [12].

Phages may protect eukaryotic cells by competing with viral adsorption and viral penetration of cells, virus-mediated cell apoptosis as well as viral replication. Phages may also induce antiviral immunity while contributing to maintaining a balanced immune response. Moreover, by inhibiting activation of NF-κB and ROS production, phages can downregulate excessive inflammatory reactions relevant in pathology and clinical course of COVID-19.

The data presented in this Commentary are often preliminary but suggest that further studies focused on the potential of phage therapy as at least an adjunct treatment of COVID-19 are warranted.

Both general and remote safety of phage therapy was confirmed [23,50–52]. Therefore, more studies including relevant clinical trials are needed to shed more light on the potential of phages to help combat the COVID-19 pandemic.
